# Pulmonary Complications following Cytoreductive Surgery and Perioperative Chemotherapy in 147 Consecutive Patients

**DOI:** 10.1155/2012/635314

**Published:** 2012-08-13

**Authors:** Vinicius Preti, David Chang, Paul H. Sugarbaker

**Affiliations:** ^1^Washington Cancer Institute, Washington Hospital Center, Washington, DC 20010, USA; ^2^Erasto Gaertner Hospital, Rua Dr. Ovande do Amaral 201, 81520-060 Curitiba, PR, Brazil; ^3^Westat, Rockville, MD 20850, USA

## Abstract

Cytoreductive surgery (CRS) with hyperthermic perioperative chemotherapy (HIPEC) has become a treatment option for selected patients with peritoneal metastases (PMs) from gastrointestinal malignancies. The purpose of this study is to evaluate our most recent data regarding pulmonary complications (respiratory distress, pleural effusion, and pneumonia) and attempt to identify risk factors associated with this management plan. This study includes the most recent 4-year experience with appendiceal and colorectal carcinomatosis patients treated in a uniform manner between January 1, 2006 and December 31, 2009. A prospective morbidity and mortality database was maintained and pulmonary adverse events were analyzed with special attention to subphrenic peritonectomy. There were 147 consecutive patients with a mean age of 49.9 years. Fourteen patients (10%) presented grades I–IV pulmonary complications for a total of 26 events. The peritonectomy of right upper quadrant was performed in 74% and right plus left in 49% of the patients. Statistically, there were no more pulmonary complications among patients submitted to peritoneal stripping of right or right and left hemidiaphragm as compared to no subdiaphragmatic peritonectomy (*P* = 1.00 and *P* = 0.58, resp.). In an analysis of 18 quantitative indicators and clinical variables with pulmonary adverse events, only blood replacement greater than six units showed a significant correlation (*P* = 0.0062). Pulmonary adverse events were observed in 10% of patients having CRS and HIPEC. Subphrenic peritonectomy was not a specific risk factor for developing these adverse events.

## 1. Introduction

Peritoneal metastases (PMs) are a cause of great morbidity and mortality in patients with gastrointestinal cancer. Problems related to the progression of PM are a frequent cause of the terminal event in these patients. A local-regional treatment that combines cytoreductive surgery (CRS) with hyperthermic perioperative chemotherapy (HIPEC) and early postoperative intraperitoneal chemotherapy (EPIC) has shown benefit in selected patients with peritoneal dissemination. This treatment has gained general acceptance for appendiceal mucinous neoplasms [1] and peritoneal mesothelioma [2] and now is finding additional applications in the management of colorectal cancer [3], gastric cancer [4], and ovarian cancer [5]. With increased experience, the morbidity and mortality have declined in several reports [6–8]. Smeenk and colleagues at The Netherlands Cancer Institute showed that over time their perioperative mortality could be diminished by 50%. Overall major morbidity was reduced from 71% between 1996 and 1998 to 34% between 2003 and 2006 [6]. Pulmonary complications are common after abdominal surgery and associated significantly with longer hospital stays [9]. The purpose of this study is to evaluate the incidence of pulmonary complications (respiratory distress, pleural effusion, and pneumonia) and to identify risk factors associated with pulmonary complications in the use of CRS and perioperative chemotherapy.

## 2. Patients and Methods

### 2.1. Patient Eligibility Criteria

This study includes our most recent 4-year experience with patients with appendiceal and colorectal PM treated in a uniform manner between January 1, 2006 and December 31, 2009. Institutional Review Board approval was obtained to collect and analyze these data. Patients with appendiceal and colorectal malignancy who received CRS combined with a standardized treatment with perioperative chemotherapy were included.

### 2.2. Cytoreductive Surgery and Hyperthermic Intraoperative Chemotherapy and Systemic Chemotherapy

The goal of surgery in these patients was to visibly clear the abdomen and pelvis of cancer nodules. This required a series of peritonectomy procedures and visceral resections [10]. Normal peritoneum or normal visceral structures were not resected. All patients received HIPEC in the operating room after the CRS but before intestinal anastomoses or repair of seromuscular tears was performed. The two drugs administered by the intraperitoneal route with heat were mitomycin C (15 mg/m^2^) and doxorubicin (15 mg/m^2^). Simultaneous intravenous 5-fluorouracil (400 mg/m^2^) and leucovorin (20 mg/m^2^) were administered as a rapid infusion over 6–8 minutes. HIPEC was given according to the Coliseum technique [10]. A heater circulator was used to maintain moderate hyperthermia within the abdomen and pelvis at 41–43°C.

### 2.3. Early Postoperative Intraperitoneal Chemotherapy

The EPIC 5-fluorouracil was withheld in patients who had a full course of oxaliplatin-based FOLFOX chemotherapy prior to surgery. The dose of EPIC 5-fluorouracil was 400 mg/m^2^/day for women and 600 mg/m^2^/day for men. It was infused via a Tenckhoff catheter over approximately 15 minutes for 4 days after surgery [10]. The dwell time for EPIC was 23 hours.

### 2.4. Perioperative Management

Patients received appropriate antibiotics within one hour prior to the abdominal incision and then throughout the cytoreductive procedure. A final dose of antibiotics was given just prior to closing the abdominal incision. No prophylactic antibiotics were given postoperatively. Patients were transferred directly to a surgical intensive care unit for monitoring and orotracheal extubation. All patients received postoperative intravenous feeding through the intrajugular vein for five postoperative days and then through a percutaneous central venous catheter (Vaxcel, Glen Falls, NY). Closed suction drains (Bard Closed Wound Suction and Silicon Drain, Covington, GA) remained in place in the abdomen and pelvis after surgery until drainage was below 50 mL per 24 hours from a single drain. Right-angle 28-French thoracostomy tubes (Deknatel, Floral Park, NY) were always used when a patient had a subphrenic peritonectomy; they were removed in the second postoperative week as drainage diminished to less than 50 mL per 24 hours.

### 2.5. Database for Morbidity/Mortality Assessment

The database was specially constructed to evaluate the adverse events including pulmonary complications (pleural effusion, respiratory distress, and pneumonia) in patients treated for PM from appendiceal and colorectal malignancy. The pulmonary adverse events which were scored grade I through grade IV are listed in Table 1.

### 2.6. Quantitative Prognostic Indicators

The extent of previous surgery was quantitated with the prior surgical score (PSS). Size and distribution of disease at the time of surgery were assessed with the peritoneal cancer index (PCI). The PCI was analyzed in three different ways: by four groups (0–10, 11–20, 21–30, and 31–39), by two groups A (0–20 versus 21+), and by two groups B (0–30 versus 31+). At the end of the cytoreductive surgery a completeness of cytoreduction score (CC-score) was recorded [11].

### 2.7. Clinical Variables

All data collection occurred on hospitalized patients; events that may have occurred after hospital discharge are not part of this analysis. Sixteen clinical variables were analyzed to assess factors predictive of pulmonary complications: gender, age (≤50 versus >50), primary cancer location (appendix versus colorectal), cancer grade (grade 1 versus grade 2-3), peritonectomy procedures (pelvic, right upper quadrant, left upper quadrant, omental bursa, anterior abdominal wall), number of peritonectomy procedures per patient (0–2 versus 3–5), visceral resections performed (omentectomy, splenectomy, rectosigmoid colon resection, right colon resection, hysterectomy, small bowel resection, transverse colon resection, and gastrectomy), visceral resections performed per patient (0–2 versus 3–7), types of anastomoses performed (esophagojejunal, small bowel, ileocolic, colocolic, and colorectal), number of anastomoses performed per patient (0–2 versus 3–5), ostomies performed (none, diverting ileostomy, and end ileostomy), blood replacement (none, 1–3 units, 4–6 units, >6 units), blood replacement (0–6 units versus >6 units), fresh frozen plasma replacement (none, 1–4 units, >4 units), time in the operating room in hours (0–6, 7–12, >12), and chemotherapy treatment (HIPEC only versus HIPEC plus EPIC).

### 2.8. Statistics

Univariate methods by Fisher's exact test, chi-square and Cochran-Mantel-Haenszel statistics and multivariate method by logistical procedure were used to assess the association between adverse pulmonary events and the subphrenic peritonectomy procedure. Those prognostic indicators and clinical variables that were significantly correlated to the outcome (*P*  value < 0.05) were then fitted into the logistic regression model for analysis of variances to assess the strength of the risk factors.

## 3. Results

### 3.1. Demographics and Clinical Features

Forty-six percent of patients were men and the mean age was 49.9 (±8.7). Peritoneal metastases from appendiceal cancer were present in 135 patients (92%) and PM from colon cancer in 12 (8%). The mean length of hospital day was 24 days. Complete cytoreduction was reported in 125 patients (85%). The right subphrenic peritonectomy was performed in 109 patients (74%) and right and left in 72 (49%). Seventy-six percent of patients required blood replacement and 46% required fresh frozen plasma transfusion. Hyperthermic perioperative chemotherapy was administered to 55.8% of patients and 44.2% received HIPEC + EPIC (Table 2).

### 3.2. Pulmonary Adverse Events

Fourteen patients (10%) presented grade II through grade IV pulmonary adverse events for a total of 23 events (Table 3).

### 3.3. Pleural Effusion

The most common event was pleural effusion with 10 events diagnosed (4.6%). Three patients were classified as grade II (diuretics required), 4 as grade III (thoracentesis required), and 3 as grade IV (chest tube insertion required).

### 3.4. Respiratory Distress

There were 9 respiratory distress events (4.2%). Two patients were classified as grade II cases (oxygen therapy or medications required), 5 as grade III (endotracheal intubation required), and 2 as grade IV (tracheostomy required). One patient died after a grade III respiratory distress followed by severe neutropenia. This was the only death among the 147 patients.

### 3.5. Pneumonia

There were 7 patients who developed pneumonia (3.2%). There were 3 grade I patients (minimal symptoms), 4 grade II patients (antibiotics and respiratory therapy required), and no grade III or IV patients (bronchoscopy or intubation required). These results are summarized in Table 3. Among the 4 grade II pneumonia patients, one presented pulmonary edema, one presented respiratory distress, and another one presented pleural effusion.

### 3.6. Analysis of Pulmonary Adverse Events by Subphrenic Peritonectomy

The patients were divided into groups with or without pulmonary complication and the impact of subphrenic peritonectomy was statistically determined. There is no difference in the incidence of pulmonary complication in the group submitted to peritoneal stripping of the right or right plus left hemidiaphragm and the group who did not have this dissection performed (Table 4).

### 3.7. Analysis of Pulmonary Adverse Events by Quantitative Prognostic Indicators and Clinical Variables

In univariate and multivariate analysis, the only risk factor was more than 6 blood units replacement. In the univariate analysis of blood replacement none, 1–3 units, 4–6 units and >6 units *P* = 0.0349. In the univariate analysis of blood replacement 0–6 units versus >6 units *P* = 0.0062 (Table 5).

In a multivariate analysis with logistic procedure, only blood replacement was identified as a risk factor for pulmonary complications (*P* = 0.0030).

## 4. Discussion

This study analyzed pulmonary complications in 147 consecutive patients at a single experienced peritoneal surface malignancy treatment center. It is the first paper to focus specifically on pulmonary complications after CRS and HIPEC. Identification of treatments-associated morbidity and mortality may help determine causation so that a reduction in complications may occur. Peritoneal metastases to the peritoneal surface of the right hemidiaphragm or right plus left hemidiaphragm were a common requirement of complete CRS. It was needed on the right in 74% of patients and right plus left in 49% of patients. Our hypothesis was that subphrenic peritonectomy would interfere with respiratory function postoperatively and thereby be associated with pulmonary adverse events. However, no relationship of peritoneal stripping of the right or right and left hemidiaphragm to pulmonary adverse events was evident.

In a recent report pulmonary complication was the second most common grade IV complications (16%) among our patients [12]. In a prior study of cytoreduction and HIPEC in nonappendiceal peritoneal metastases patients, it was the most common grade IV adverse event at 26% [13]. The incidence of grade I through IV pneumonia, pleural effusion, and respiratory distress of 10% is reported in this paper. Kusamura related 12% incidence of major complications and the most common cause of morbidity was anastomotic leak or intestinal perforation. Their second most common complication was the pulmonary [14].

Pleural effusion is a relatively common event described in many reports and it could be due to several factors. The stripping of the diaphragmatic peritoneum elicits a mechanical and thermal injury to the muscle. This trauma would promote with fluid access to the thorax from the abdomen of chemotherapy solution during HIPEC. Chéreau et al. showed a higher incidence of pleural effusion and other pulmonary complications in a group of ovarian cancer patients submitted to peritoneal diaphragmatic resection; they reported a greater number of patients requiring pleural drainage [15]. In this report, opening the pleura was required because of the carcinomatosis infiltration of the diaphragm; systematic pleural drainage was not performed routinely in these patients. Dowdy et al. also showed pleural effusion as their most common complication, with an incidence of 30% among 56 patients [16]. Stephens and colleagues related an incidence of 3% of pleural effusion among 200 patients submitted to peritonectomy and HIPEC [17]. The only predictor for the development of postoperative pleural effusion was entry into the pleural space at the time of diaphragm peritonectomy. Pleural drainage was routine in all our patients in an attempt to avoid pleural effusion. Nevertheless, pleural effusion remained the second most common respiratory event. In our patients, there is no statistical correlation that showed that stripping the diaphragm is a risk factor for pulmonary adverse events.

Postoperative infection is a high-risk factor in patients submitted to peritonectomy procedures and it is fundamental to recognize an infectious process at an early stage [18]. Among the infectious adverse effects, pneumonia ranged from 3.5 to 6.6% in recent series [13, 19]. In the past, Schmidt reported this incidence had reached up to 10% [20]. In this series, pneumonia occurred in 3.2% of our patients.

The morbidity and mortality have been reduced in several reports with increasing experience with CRS and HIPEC. Smeenk and colleagues reported a decrease in morbidity from 71.2 to 34% in an 8-year period in a multicentric analysis [6]. Muller and colleagues showed that it was possible to reduce the adverse effects by reducing inflammatory response, with intraoperative fluid restriction, intensified hyperglycemia management, and reducing the blood loss [21]. Mohamed and Moran demonstrated the importance of a learning curve in CRS and HIPEC to reduce the incidence of adverse effects. They defended the importance of teamwork and the presence of 2 experienced surgeons to support each other in the management of a multidisciplinary team and to confer regarding the rationale, indications, and the morbidity associated with this procedure. It is possible to perform peritonectomy and HIPEC with morbidity and mortality rates in line with those of other major oncologic procedures [7].

## Figures and Tables

**Table 1:** Classification of pulmonary adverse event by grade.

Adverse event	Grade I	Grade II	Grade III	Grade IV
Respiratory distress	Mild symptoms	Oxygen therapy or medications required	Endotracheal intubation	Tracheostomy required
Pleural effusion	Asymptomatic	Diuretics required	Thoracentesis required	Compromised, chest tube insertion
Pneumonia	Minimal symptoms	Antibiotics and respiratory therapy	Bronchoscopy	Intubation required

**Table 2:** Demographic and clinical features.

Patients	
Male	68 (46%)
Female	79 (54%)
Age (years)	
Mean ± standard deviation	49.9 (8.7%)
Median	51 (27%)
Range	23–64
Primary cancer diagnosis	
Appendix	135 (92%)
Colorectal	12 (8%)
Completeness of cytoreduction	
Complete	125 (85%)
Incomplete	22 (15%)
Subphrenic peritonectomy	
Right	109 (74%)
Right and left	72 (49%)
Blood products	
None	39 (26.5%)
1–3 units	68 (46.3%)
4 or more	40 (27.2%)
Fresh frozen plasma	
None	80 (54%)
1–4 units	51 (34.7%)
5 or more	16 (10.9%)
Chemotherapy treatments	
HIPEC	82 (55.8%)
HIPEC + EPIC	65 (44.2%)

**Table 3:** Pulmonary adverse events grade I through grade IV. There was a total of 26 pulmonary adverse events in 14 patients.

Organ System	Absolute number/%	Grade I	Grade II-symptomatic and medical treatment	Grade III-invasive intervention	Grade IV-ICU care or return to operating room
Pleural effusion	10/4.6%	Asymptomatic 0%	Diuretics required 3/1.4%	Thoracentesis required 4/1.8%	Compromised, chest tube insertion 3/1.4%
Respiratory distress	9/4.2%	Mild symptom 0%	Oxygen therapy or medications required 2/0.9%	Endotracheal intubation 5/2.3%	Tracheostomy required 2/0.9%
Pneumonia	7/3.2%	Minimal symptoms 3/1.4%	Antibiotics and respiratory therapy 4/1.8%	Bronchoscopy 0%	Intubation required 0%

**Table 4:** Analysis of pulmonary adverse events (pleural effusion, respiratory distress, and pneumonia) by presence versus absence of subdiaphragmatic peritonectomy. *P* value based on Fisher's exact test.

		No pulmonary complication (*N* = 133)	Pulmonary complication occurred (*N* = 14)	Total	*P* value
RUQ + LUQ	No	69 (92%)	6 (8%)	75	*0.5826*
	Yes	64 (89%)	8 (11%)	72	

RUQ	No	35 (92%)	3 (8%)	38	*1.0000*
	Yes	98 (90%)	11 (10%)	109	

LUQ: left upper quadrant, RUQ: right upper quadrant.

**Table 5:** Impact of quantitative prognostic indicators and clinical variables on pulmonary adverse events in 147 consecutive patients.

	Pulmonary			Pulmonary	
	Events I–IV			Events I–IV	
	Univariate analysis			Multivariate analysis	
	Yes *N* = 14	No *N* = 133	*P* value^∗^/OR (95% CI)	Odds ratio	*P* value
Gender					
Male	6	62	0.7884		NT^∗∗^
Female	8	71	1.2 (0.4, 3.5)		
Age					
≤50 year	7	65	0.9360		NT
>50 year	7	68	1.0 (0.3, 2.9)		
Location					
Appendix	13	122	1.0000		NT
Colorectal	1	11	0.9 (0.1, 7.1)		
Grade					
Grade 1	4	57	0.3021		NT
Grade 2–4	10	76	1.9 (0.6, 6.3)		
Prior surgical score					
0–2	13	120	1.0000		NT
3–5	1	13	0.7 (0.1, 5.9)		
Peritoneal cancer index (4 groups)					
0–10	1	30	reference		NT
11–20	4	36	0.3779		
			3.3 (0.4, 31.4)		
21–30	5	48	0.4060		
			3.1 (0.3, 28.1)		
31–39	4	19	0.1512		
			6.3 (0.7, 60.9)		
Peritoneal cancer index (2 groups A)					
0–20	5	66	0.3218		NT
21+	9	67	1.8 (0.6, 5.6)		
Peritoneal cancer index (2 groups B)					
0–30	10	114	0.2359		NT
31+	4	19	2.4 (0.7, 8.4)		
Completeness of cytoreduction					
Complete	10	115	0.2273		NT
Incomplete	4	18	2.6 (0.7, 9.0)		
Peritonectomy procedure					
Pelvic	13	110	0.4679		NT
			0.4 (0.1, 2.9)		
Right upper quadrant	11	98	1.0000		NT
			0.8 (0.2, 2.9)		
Left upper quadrant	8	64	0.5206		NT
			0.7 (0.2, 2.1)		
Omental bursa	10	60	0.0608		NT
			0.3 (0.1, 1.1)		
Anterior abd. wall	6	44	0.5553		NT
			0.7 (0.2, 2.0)		
Peritonectomy procedure per patient					
0–2	3	52	0.1938		NT
3–5	11	81	2.4 (0.6, 8.8)		
					
Visceral resections performed					
Omentectomy	14	130	1.0000		NT
			NC^∗∗^		
Splenectomy	11	73	0.0885		NT
			0.3 (0.1, 1.2)		
Rectosigmoid colon	7	50	0.3648		NT
			0.6 (0.2, 1.8)		
Right colon resection	7	63	0.8512		NT
			0.9 (0.3, 2.7)		
Hysterectomy	4	43	1.0000		NT
			1.2 (0.4, 4.0)		
Small bowel resection	2	27	0.7375		NT
			1.5 (0.3, 7.2)		
Transverse colon resection	3	17	0.4078		NT
			0.5 (0.1, 2.1)		
Gastrectomy	0	4	1.0000		NT
			NC		
Visceral resections performed per patient					
0–2	5	51	0.8471		NT
3–7	9	82	1.1 (0.4, 3.5)		
Anastomoses performed					
Esophagojejunal	0	2	1.0000		NT
			NC		
Small bowel	1	21	0.6945		NT
			2.4 (0.3, 19.6)		
Ileocolic	1	28	0.3038		NT
			3.5 (0.4, 27.6)		
Colocolic	0	3	1.0000		NT
			NC		
Colorectal	5	51	0.8471		NT
			1.1 (0.4, 3.5)		
Anastomoses performed per patient					
0–2	14	125	1.0000		NT
3–5	0	8	NC		
Ostomies performed					
None	9	95	reference		
Diverting ileostomy	3	27	0.7305		NT
			1.2 (0.3, 4.6)		
End ileostomy	2	11	0.3518		NT
			1.9 (0.4, 10.0)		
Blood replacement					
None	5	34	reference		
Blood 1–3	2	66	0.0966		NT
			0.2 (0.04, 1.1)		
Blood 4–6	4	31	1.0000		NT
			0.9 (0.2, 3.6)		
Blood >6	3	2	0.0349	10.2 (1.4, 76.9)	*0.0030*
			10.2 (1.4, 76.9)		
					
Blood replacement					
Blood 0–6	11	131	Reference		
Blood >6	3	2	0.0062
			17.9 (2.7,118.5)
Fresh frozen plasma replacement					
None	7	73	reference		
Plasma 1–4	4	47	1.0000		NT
			0.9 (0.2, 3.2)		
Plasma >4	3	13	0.3627		NT
			2.4 (0.6, 10.5)		
Time in operating room (hours)					
0–6	0	10	Reference		
7–12	12	112	0.5986		NT
			NC		
>12	2	11	0.4862		NT
			NC		
Chemotherapy treatment					
HIPEC only	5	74	0.2128		NT
HIPEC plus EPIC	8	57	2.1 (0.6, 6.7)		
Unknown	2	1			

^∗^Pearson Chi-square or Fisher's exact test if sparse distribution.

^∗∗^NC means not calculated due to 0 count in any of the cells.

^∗∗∗^NT means not tested in multivariate modeling due to nonsignificant univariate test.

## References

[1] Sugarbaker PH (2006). New standard of care for appendiceal epithelial neoplasms and pseudomyxoma peritonei syndrome?. *Lancet Oncology*.

[2] Yan TD, Welch L, Black D, Sugarbaker PH (2007). A systematic review on the efficacy of cytoreductive surgery combined with perioperative intraperitoneal chemotherapy for diffuse malignancy peritoneal mesothelioma. *Annals of Oncology*.

[3] Chua TC, Liauw W, Morris DL, Sugarbaker PH (2012). Colorectal cancer: prevention and treatment of peritoneal metastases. *Cytoreductive Surgery and Perioperative Chemotherapy For Peritoneal Surface Malignancy: Textbook and Video Atlas*.

[4] Glehen O, Yonemura Y, Sugarbaker PH, Sugarbaker PH (2012). Prevention and treatment of peritoneal metastases from gastric cancer. *Cytoreductive Surgery and Perioperative Chemotherapy For Peritoneal Surface Malignancy: Textbook and Video Atlas*.

[5] Helm CW, Sugarbaker PH (2012). Epithelial ovarian cancer with peritoneal metastases. *Cytoreductive Surgery and Perioperative Chemotherapy For Peritoneal Surface Malignancy: Textbook and Video Atlas*.

[6] Smeenk RM, Verwaal VJ, Zoetmulder FAN (2007). Learning curve of combined modality treatment in peritoneal surface disease. *British Journal of Surgery*.

[7] Mohamed F, Moran BJ (2009). Morbidity and mortality with cytoreductive surgery and intraperitoneal chemotherapy: the importance of a learning curve. *Cancer Journal*.

[8] Ahmad SA, Kim J, Sussman JJ (2004). Reduced morbidity following cytoreductive surgery and intraperitoneal hyperthermic chemoperfusion. *Annals of Surgical Oncology*.

[9] Lawrence VA, Hilsenbeck SG, Mulrow CD, Dhanda R, Sapp J, Page CP (1995). Incidence and hospital stay for cardiac and pulmonary complications after abdominal surgery. *Journal of General Internal Medicine*.

[10] Sugarbaker PH (2009). Comprehensive management of peritoneal surface malignancy using cytoreductive surgery and perioperative intraperitoneal chemotherapy: the Washington Cancer Institute approach. *Expert Opinion on Pharmacotherapy*.

[11] Jacquet P, Sugarbaker PH (1996). Current methodologies for clinical assessment of patients with peritoneal carcinomatosis. *Journal of Experimental and Clinical Cancer Research*.

[12] Sugarbaker PH, Van der Speeten K, Stuart OA, Chang D, Mahteme H, Sugarbaker PH Patient- and treatment-related variables, adverse events and their statistical relationship for treatment of peritoneal metastases. *Cytoreductive Surgery and Perioperative Chemotherapy For Peritoneal Surface Malignancy: Textbook and Video Atlas*.

[13] Yan TD, Zappa L, Edwards G, Alderman R, Marquardt CE, Sugarbaker PH (2007). Perioperative outcomes of cytoreductive surgery and perioperative intraperitoneal chemotherapy for non-Appendiceal peritoneal carcinomatosis from a prospective database. *Journal of Surgical Oncology*.

[14] Kusamura S, Younan R, Baratti D (2006). Cytoreductive surgery followed by intraperitoneal hyperthermic perfusion: analysis of morbidity and mortality in 209 peritoneal surface malignancies treated with closed abdomen technique. *Cancer*.

[15] Chéreau E, Ballester M, Selle F (2009). Pulmonary morbidity of diaphragmatic surgery for stage III/IV ovarian cancer. *An International Journal of Obstetrics and Gynaecology*.

[16] Dowdy SC, Loewen RT, Aletti G, Feitoza SS, Cliby W (2008). Assessment of outcomes and morbidity following diaphragmatic peritonectomy for women with ovarian carcinoma. *Gynecologic Oncology*.

[17] Stephens AD, Alderman R, Chang D (1999). Morbidity and mortality analysis of 200 treatments with cytoreductive surgery and hyperthermic intraoperative intraperitoneal chemotherapy using the coliseum technique. *Annals of Surgical Oncology*.

[18] Capone A, Valle M, Proietti F, Federici O, Garofalo A, Petrosillo N (2007). Postoperative infections in cytoreductive surgery with hyperthermic intraperitoneal intraoperative chemotherapy for peritoneal carcinomatosis. *Journal of Surgical Oncology*.

[19] Glehen O, Osinsky D, Cotte E (2003). Intraperitoneal chemohyperthermia using a closed abdominal procedure and cytoreductive surgery for the treatment of peritoneal carcinomatosis: morbidity and mortality analysis of 216 consecutive procedures. *Annals of Surgical Oncology*.

[20] Schmidt U, Dahlke MH, Klempnauer J, Schlitt HJ, Piso P (2005). Perioperative morbidity and quality of life in long-term survivors following cytoreductive surgery and hyperthermic intraperitoneal chemotherapy. *European Journal of Surgical Oncology*.

[21] Müller H, Hahn M, Weller L, Simsa J (2008). Strategies to reduce perioperative morbidity in cytoreductive surgery. *Hepato-Gastroenterology*.

